# Effect of sheep grazing on seed circulation on the Loess Plateau

**DOI:** 10.1002/ece3.8368

**Published:** 2021-11-17

**Authors:** Shu‐Lin Wang, An Hu, Fu‐Jiang Hou

**Affiliations:** ^1^ State Key Laboratory of Grassland Agro‐Ecosystems Key Laboratory of Grassland Livestock Industry Innovation Ministry of Agriculture Lanzhou China; ^2^ College of Pastoral Agriculture Science and Technology Lanzhou University Lanzhou China

**Keywords:** dung seed bank, semiarid area, soil seed bank, stocking rate, tan sheep

## Abstract

In grazing ecosystems, mature seeds fall directly to the soil to form the soil seed bank (SSB), or are ingested by grazing livestock to become part of the dung seed bank (DSB; i.e., seed circulation). Both the SSB and DSB form the basis for the natural regeneration of vegetation. However, little is known about the relationships between the SSB, DSB, and aboveground vegetation (AGV) community under different stocking rates (SRs). This study investigated the relationships between the SSB, seeds in Tan sheep (*Ovis aries*) dung, and AGV at different SRs (0, 2.7, 5.3, and 8.7 sheep ha^–1^) in a semiarid region of the Loess Plateau in China. We found that Tan sheep grazing increased the species richness heterogeneity of grassland vegetation, and negatively influenced the density of AGV. Under natural conditions, 17 species from soil‐borne seeds and 10 species from Tan sheep dung germinated. There was low species similarity between the soil and DSBs and AGV. Sheep SR and the seed banks (soil and dung) were negatively correlated with AGV. Seeds are cycled from herbage to livestock to soil during cold season grazing; the seasonal nature of this seed dispersal is an adaptation to harsh, semiarid environments. Increased seed bank diversity under sheep grazing facilitates grassland regeneration on the Loess Plateau, similarly to other semiarid regions globally.

## Graphical abstract

Mature seeds can fall directly onto the soil to form the soil seed bank or become part of the dung seed bank through livestock grazing. Seeds in the feces eventually are incorporated into the soil, contributing to the soil seed bank. Seed cycling was depressed at higher grazing intensities. Sheep grazing disturbance increases the diversity of seed banks and assists in the propagation and regeneration of vegetation on the Loess Plateau and in other semi‐arid regions of the world.

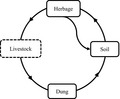

## 1 INTRODUCTION

The soil seed bank (SSB) is the basis for the natural regeneration of vegetation, especially in severely eroded or degraded ecosystems (Baskin & Baskin, 1978). In rangelands, the degree of heterogeneity among SSBs is strongly influenced by livestock pressure through selective grazing and grazing‐related environmental impacts (Kassahun et al., 2009; Solomon et al., 2006). The diversity of seeds in disturbed habitats is determined by the original plant populations, propagule production, and soil seed reserves (Grime, 1979), all of which are affected by ecological perturbations. The quality of the SSB declines as a function of time as vegetation is destroyed (Bakker et al., 1996). Therefore, the SSBs of arid and semiarid ecosystems are highly variable over space and time, and are linked to factors that influence seed production, mortality, and spatial distribution (Kemp, 1989). Drought cycles (Msangi, 2004) and overgrazing (Klintenberg & Seely, 2004) are the primary drivers of dryland degradation, and together can result in broad‐scale restructuring of vegetation and seed resources. Therefore, understanding the impact of grazing‐related environmental changes on SSBs and the relationship between seed banks and aboveground vegetation (AGV) diversity are critical to inform grassland management, conservation, and restoration (Kassahun et al., 2009).

When mature plant seeds are consumed by foraging livestock, some of these seeds survive passage through the digestive tract and are ultimately deposited in dung. These viable seeds in herbivore feces constitute the dung seed bank (DSB; Wang & Hou, 2021; Wang, Hu, et al., 2019), which defined as a subset of the SSB and is an important source of vegetation renewal. The structure of the DSB depends on rangeland composition and selective feeding by livestock (Wang & Hou, 2021). The DSB is a special form of the SSB, as once dung decomposes, seeds in the feces are eventually incorporated into the soil, contributing to the SSB. Therefore, the DSB is a key factor that determines pasture seed dispersal, SSB composition, and seedling density, inducing changes in grassland vegetation composition (Elisabeth & Han, 2003; Wang et al., 2017). A variety of germinable plant seeds accumulates in feces; hence, the DSB is also an important driving force for promoting the formation of grassland patches (Myers et al., 2004). Fecal sedimentation, dung‐borne seed germination, and seedling establishment in feces increase the similarity of plant communities between different types of grazed grasslands, and fosters diversity among grassland plants within local communities (Malo & Suárez, 1995). Therefore, researching the DSB’s composition, size, and ecological characteristics is essential for studies in grazing ecology (D'Hondt & Hoffmann, 2015).

A number of studies have indicated that seedling emergence and growth are promoted by the organic matter and nutrients in livestock dung (Nchanji & Plumptre, 2003; Traveset et al., 2001; Woldu & Saleem, 2000). It is assumed that seed ingestion by livestock increases plant species richness and influences the large‐scale spatial community composition of grazed ecosystems by intensifying the intercommunity seed flow. In this way, the dynamics and species richness of these grazed ecosystems can be substantially affected by seed quantity and the range of seed species dispersed by herbivores (Pakeman et al., 2002). However, the relationship between the DSB and SSB under different stocking rates (SRs) remains unclear, as does the mechanism by which this relationship is maintained (Albert et al., 2015).

**FIGURE 1:**
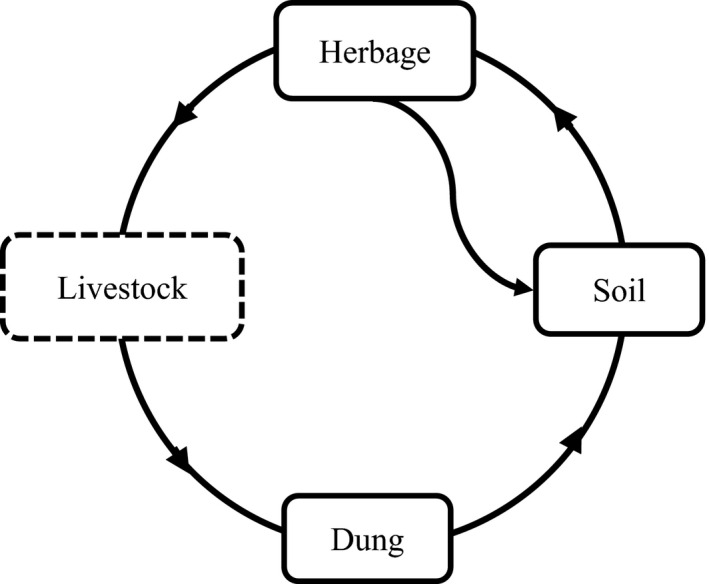
The path of seed in grazed grassland systems

Plants in arid and semiarid environments are primarily propagated by seeds (Gutterman, 1994). Mature seeds fall directly to the soil to form the SSB or are ingested by grazing livestock to become part of the DSB (Figure 1). Both the SSB and DSB form the basis for the natural regeneration of vegetation, especially in severely disturbed and degraded ecosystems (Hu et al., 2019; Kapás et al., 2020; Wang, Hu, et al., 2019). Previous studies indicated that SR had no effect on seed density and species richness in sheep dung or subsequent seedling diversity due to reduced forage seed intake as SR increased (Langlands & Bennett, 1973; Wang, Hu, et al., 2019). However, grazing can increase the richness and diversity of the SSB by increasing the richness and diversity of the AGV (Hu et al., 2019). However, the effect of SR on seed circulation process in grazing ecosystems remains unclear (Auffret & Plue, 2014).

Our previous work (Hu et al., 2019; Wang, Hu, et al., 2019) showed that Tan sheep (*Ovis aries*) dung plays an important role in seed dispersal, species richness, and species diversity on dry grasslands. This study aimed to assess the effects of different Tan sheep SR (0, 2.7, 5.3, and 8.7 sheep ha^−1^) on seed cycling by determining the relationships between the SR, SSB, Tan sheep DSB, and AGV diversity over two successive years (2016 and 2017) in a typical Loess Plateau grassland. First, we investigated the relationship between AGV diversity and the DSB and SSB under different SR, and then we evaluated the correlations between pasture disturbance caused by sheep grazing and the seed bank species richness and density. We hypothesized that the SR could directly affect the 2017 AGV composition, or indirectly affect the 2017 AGV characteristics as a consequence of changes in the size and composition of the 2016 SSB and/or DSB. The results enhance our understanding of the ecological significance of the SSB/DSB for grassland development and the influence mechanisms of grazing on seed circulation in grassland ecosystems.

## 2 MATERIAL AND METHODS

### 2.1 Study area

We selected a study site located in a typical steppe on Lanzhou University's Huanxian Grassland Agriculture Research Station [Huan County, Eastern Gansu Province, north‐western China (37.14°N, 106.84°E; 1650 m elevation)]. Only two seasons are defined for this area: warm and cold. In this region, over 70% of the annual precipitation occurs between July and September. In the grasslands, flowering occurs predominantly during the late warm season (July to August), with seeds reaching maturity during the early cold season (October to November). Thus, the DSB and SSB are principally replenished during cold‐season grazing. The soil of this region is classified as a sandy, free‐draining loess, and the rangeland is classified as a typical temperate steppe (Hou et al., 2002; Hu et al., 2019).

### 2.2 Experimental design

A flatland area (c. 5 ha) was selected and divided into nine paddocks of equal size (100 × 50 m^2^). A rotational grazing system was established using 4‐month‐old castrated male Tan sheep, with three SR (each SR was replicated three times) of 2.7, 5.3, and 8.7 sheep ha^–1^ (*n* = 4, 8, and 13 sheep per paddock, respectively; Chen et al., 2010; Chen et al., 2015; Hu et al., 2019; Wang, Hu, et al., 2019; Li et al., 2021). SR were based on data for long‐term grass yield, livestock weight, and feed intake (Wang, Hu, et al., 2019). Sheep of a given SR were rotationally grazed in three randomly selected paddocks from mid‐November through the end of December each year since 2000 (Hu et al., 2019; Wang, Hu, et al., 2019). In the first cycle (24 days), sheep were shifted between these three paddocks every 8 days. In the second cycle (21 days), the sheep shifted paddocks every 7 days. One 5 × 5 m^2^ fenced area was set up with a 50‐m boundary along each paddock's midline to serve as a control, nongrazing plot (CK, 9 in total).

### 2.3 Sampling

#### 2.3.1 AGV community

Herbaceous species were identified during the peak growth season (mid‐August of 2016 and 2017), when AGV had the highest species richness (29 species). Six quadrats (1 × 1 m^2^) were laid in the middle of each of the three CK plots and grazing paddocks. Plant species richness (number of plant species) and species density (number of individuals of each species within the entire 1 m^2^ area) were recorded for each quadrat.

#### 2.3.2 DSB sampling

Dung samples were collected from each of the nine grazing paddocks (late December 2016). Fecal pellets were pooled to obtain a sample of c. 2 kg fresh weight (65.54% water content) per plot. Each dung sample was split into six equal subsamples of 300 g. Fifty‐four subsamples (6 subsamples × 3 SR × 3 replicates) were placed in a clearly marked canvas bag and transported to the laboratory. Each subsample was dried at 35°C for ~72 h in a drying oven to prevent decay and premature seed germination. Importantly, drying at this temperature does not substantively affect the germination potential of seeds in dung (Wang, Hu, et al., 2019). All dried dung samples were weighed and then stored in the dark at room temperature.

#### 2.3.3 SSB sampling

The SSB was sampled at the same time as the DSB, that is, at the end of grazing and after the time of natural seed production in the pasture. These samples served to estimate the proportion of ungerminated viable seeds remaining in the seed bank after deposition. In each of the nine grazing paddocks, twelve core samples (9 cm diameter ×10 cm deep) were taken along three lines spaced 10 m apart; each line consisted of four samples that were spaced 20 m apart. In each of the three CK plots, four samples were taken along two lines spaced 2 m apart; each line consisted of two samples that were spaced 2 m apart. Samples from the same paddock or CK plot were pooled, mixed, and then split into six equal subsamples, for a total of 72 [(6 subsamples × 3 SR × 3 replicates) + (6 subsamples × 3 CK)]. All subsamples (soil only) were vernalized outdoors under freezing temperatures for two months before performing the germination assays.

### 2.4 Germination assay

DSB and SSB seed germination was observed under natural conditions in the Huanxian Research Station yard (He et al., 2020). One hundred grams of dried dung (gently broken up to avoid damage to seeds) mixed with ~50 g sterilized sand and potted in a 2‐cm layer over 5 cm of vermiculite, then covered with 2 cm of soil. Ten pots containing only sterilized sand and vermiculite were placed alongside the dung/soil pots as controls for wind‐blown seeds or other forms of seed contamination. All pots (136 total: 54 dung pots + 72 soil pots + 10 control) were irrigated twice a day (i.e., in the morning and afternoon, except on rainy days) from January through June 2017. The experiment was ended after 6 months, when no substantive new germination had been detected for 2 weeks (Malo, 2000). Emerging seedlings were recorded and removed as soon as they could be identified, or were transplanted into separate pots for later identification (Wang, Hu, et al., 2019). Whenever seedlings were removed, the dung/sand mix or soil sample was stirred to facilitate additional germination of buried seeds.

### 2.5 Statistical analysis

Data were analyzed using SPSS (version 26.0; SPSS, Inc.). All data were checked for a normal distribution using a Shapiro–Wilk test. The DSB and SSB species richness and seedling density were log_10_‐transformed to achieve normality and homogeneity of variances. We set SR as a fixed effect. We compared the plant species richness between paired controls and grazing paddocks using a nonmetric multidimensional scaling analysis (PCORD 5.0) with a Raup–Crick dissimilarity matrix (Plue et al., 2020; Raup & Crick, 1979). Data on the Raup–Crick dissimilarity index, as well as the Jaccard similarity coefficient (*J*) index, were analyzed using one‐way ANOVA with least significant difference (LSD) for multiple comparisons. The *J* index was used to test for species composition similarities at various SRs between the SSB and DSB (2016) and between the SSB/DSB and AGV (2016 and 2017). The *J* index is defined as:

(1) $$J=\frac{a}{b}$$

where *a* is the number of common species (i.e., richness) between two groups at the same SR, and *b* is the total number of species.

Structural equation modeling (SEM) was used to estimate the contribution of the SR to changes in AGV (based on species density in 2016 and 2017) and the SSB and DSB. A chi‐squared test was used to evaluate the model's fit. The model was considered to have a good fit when 0 ≤ χ^2^/df ≤ 2 and .05 < *p* ≤ 1. A large *p* value (>.05) indicated that the data's covariance structure did not differ significantly from the expected model (Grace, 2006). SEM analyses were performed using AMOS 22 (Arbuckle, 2010).

## 3 RESULTS

### 3.1 Effect of grazing on species composition

**FIGURE 2:**
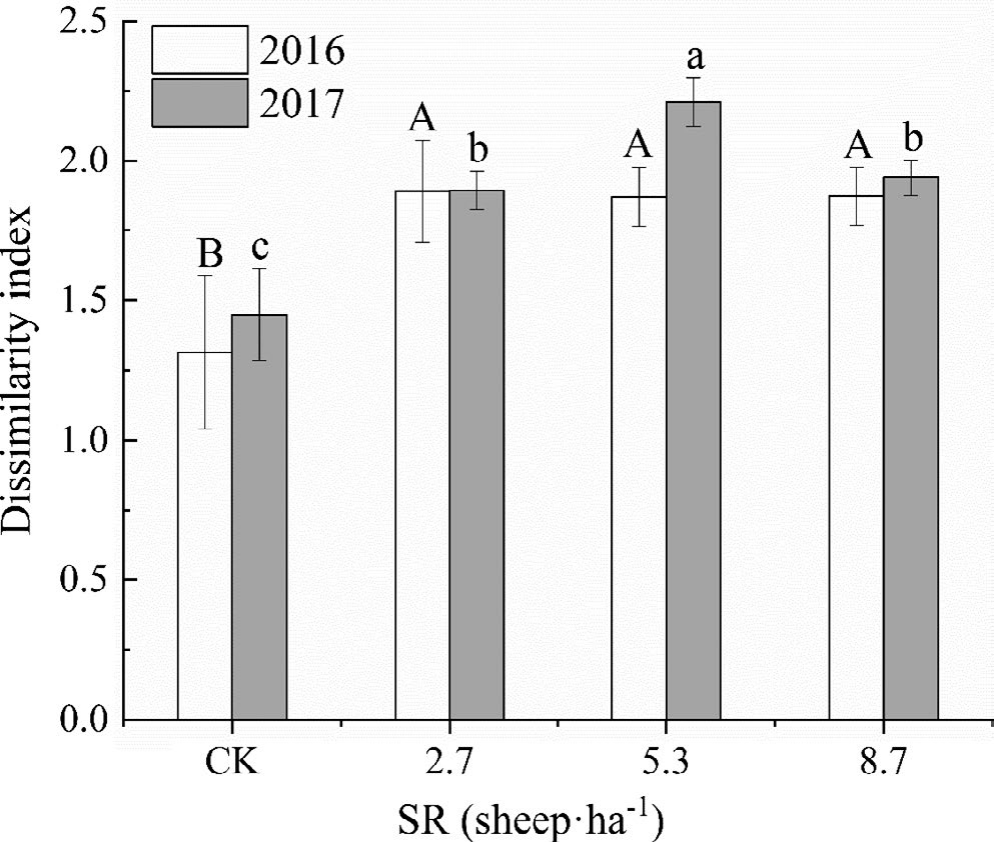
Dissimilarity index of plant species composition among paddocks affected by SR in 2016 and 2017. Different capital letters show significant differences between SR in 2016 (*p* < .05); different lowercase letters show significant differences between SR in 2017 (*p* < .05). CK, control (non‐grazing); error bars, standard error; SR, stocking rate

In both 2016 and 2017, all of the grazed paddocks had a significantly higher dissimilarity index than did CK (*F*_3,20_ = 2.90; *p* < .05; Figure 2), indicating that grazing significantly changed the species composition of grassland vegetation. In 2016, there was no significant difference in the dissimilarity index for species composition among grazing paddocks with different SR (*F*_3,20_ = 4.87; *p* > .05); however, in 2017, the dissimilarity index was significantly higher at SR 5.3 than at SR 2.7 and 8.7 (*F*_3, 20_ = 2.88; *p* < .05).

### 3.2 SSB germination

Seventeen herb species germinated from the SSB samples, representing nine families (Table 1). Species that were unique to only one SR included *Plantago minuta* Pall. (SR = 5.3 sheep ha^−1^); *Ixeridium gracile* (DC.) Shih (8.7 sheep ha^−1^); *Cleistogenes songorica* (Roshev.) Ohwi (2.7 sheep ha^−1^); *Gueldenstaedtia verna* (Georgi) Boriss. (2.7 sheep ha^−1^); and *Chenopodium glaucum* L. (2.7 sheep ha^−1^). Nine species were found only in grazed paddocks. Two species (*Lespedeza bicolor* Turcz. and *Artemisia capillaris* Thunb.) were found in all paddocks. Among the species identified, *A*. *capillaris* contributed the most to the total SSB density (51.1%–76.1%), although its density decreased with increasing SR.

**TABLE 1:** SSB species and germination density (mean ± SE seeds m^–2^) at different SRs in 2017

Family	Species	SR (sheep ha^–1^)			
		0	2.7	5.3	8.7
Asteraceae	*Artemisia capillaris* Thunb.	1416 ± 120	1075 ± 120	944 ± 164	603 ± 69
	*Heteropappus altaicus* (Willd.) Novopokr.	—	—	52 ± 43	79 ± 0
	*Ixeridium gracile* (DC.) Shih	—	—	—	26 ± 21
Gramineae	*C*. *songorica* (Roshev.) Ohwi	—	26 ± 21	—	—
	*Cleistogenes squarrosa* (Trin.) Keng	—	—	52 ± 21	26 ± 21
	*Eragrostis pilosa* (L.) Beauv.	—	—	26 ± 21	26 ± 21
	*Stipa bungeana* Trin.	131 ± 21	236 ± 98	78 ± 45	79 ± 0
Leguminosae	*Astragalus melilotoides* Pall.	26 ± 21	—	26 ± 21	26 ± 21
	*G*. *verna* (Georgi) Boriss.	—	26 ± 21	—	—
	*Lespedeza bicolor* Turcz.	78 ± 45	26 ± 21	52 ± 21	52 ± 21
Rosaceae	*Potentilla bifurca* L.	52 ± 43	—	52 ± 21	26 ± 21
	*Potentilla multifida* L.	105 ± 86	—	52 ± 21	79 ± 0
Mazaceae	*Dodartia orientalis* L.	26 ± 21	—	26 ± 21	78 ± 78
Plantaginaceae	*P*. *minuta* Pall.	—	—	26 ± 21	—
Chenopodiaceae	*C*. *glaucum* L.	—	26 ± 21	—	—
Brassicaceae	*Neotorularia humilis* (C. A. Meyer) O. E. Schulz	—	26 ± 21	—	52 ± 21
Boraginaceae	*Lappula deserticola* C. J. Wang	26 ± 21	—	52 ± 21	26 ± 21

Note

—, species not found at given SR. Latin names were obtained from the Flora Reipublicae Popularis Sinicae (http://www.iplant.cn/frps).

### 3.3 DSB germination

The average seedling density from DSB subsamples was 0.72 seedlings·g^–1^ dung. Five species germinated from the dung samples collected from grazing paddocks. Eight, seven, and six plant species germinated from dung at SR 2.7, 5.3, and 8.7, respectively (Table 2).

**TABLE 2:** Seedling density (mean ± SE seedling g^−1^) of herbaceous species identified from the sheep DSB at different SRs in 2017

Family	Species	SR (sheep ha^−1^)		
		2.7	5.3	8.7
Asteraceae	*A. capillaris*	0.43 ± 0.14	0.42 ± 0.12	0.37 ± 0.11
Gramineae	*C. songorica*	0.03 ± 0.00	0.02 ± 0.00	—
	*E. pilosa*	0.03 ± 0.00	0.07 ± 0.02	0.02 ± 0.00
	*Leymus secalinus* (Georgi) Tzvel.	0.02 ± 0.00	—	—
Leguminosae	*G. verna*	0.02 ± 0.00	0.02 ± 0.00	0.05 ± 0.00
	*L. bicolor*	0.04 ± 0.00	0.04 ± 0.00	0.04 ± 0.01
Rosaceae	*P. bifurca*	0.05 ± 0.00	—	—
	*P. multifida*	—	0.02 ± 0.00	—
Mazaceae	*D. orientalis*	—	—	0.05 ± 0.00
Chenopodiaceae	*C. glaucum*	0.11 ± 0.05	0.10 ± 0.02	0.06 ± 0.01

Note

—, species not found at given SR.

### 3.4 Similarities between the SSB, DSB, and AGV

**FIGURE 3:**
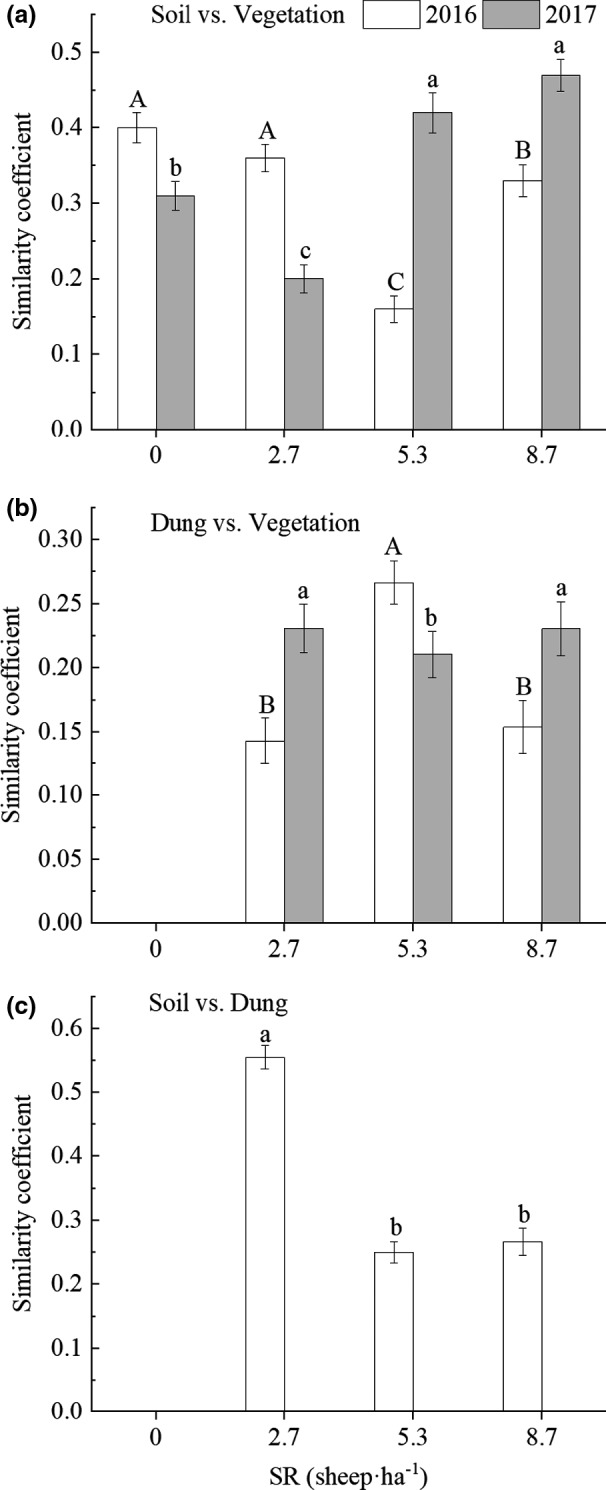
Similarity between SSB and aboveground vegetation (a), between DSB and AGV (b), and between SSB and DSB (c) at different SR in 2016 and/or 2017. Different capital letters show significant differences between SR in 2016 (*p* < .05); different lowercase letters show significant differences between SR in 2017 (*p* < .05). SSB: soil seed bank; DSB: dung seed bank; AGV, aboveground vegetation; SR, stocking rate; error bars: standard error

*J* values between SSB composition and AGV at different SR were <0.5 for both grazed and CK paddocks. In 2016, the similarity between the SSB and AGV was significantly higher at SR 0 and 2.7 than at SR 5.3 and 8.7 (*F*_3, 20_ = 5.99; *p* < .05; Figure 3a). In 2017, the similarity between the SSB and AGV was significantly higher at SR 5.3 and 8.7 than at SR 0 and 2.7 (*F*_3, 20_ = 4.74; *p* < .05; Figure 3a).

*J* values between DSB composition and AGV at different SR were <0.5 for both grazed and CK paddocks (Figure 3b). In 2016, the similarity between the DSB and AGV was significantly higher at SR 5.3 than at SR 2.7 and 8.7 (*F*_3,32_ = 2.92; *p* < .05). In 2017, the similarity between the DSB and AGV was significantly higher at SR 2.7 and 8.7 than at SR 5.3 (*F*_3,32_ = 2.69; *p* < .05; Figure 3b).

In 2016, the similarity between the SSB and DSB was significantly higher at SR 2.7 than at SR 5.3 and 8.7 (*F*_3,32_ = 4.53; *p* < .05; Figure 3c).

### 3.5 Relationships between the SSB, DSB, AGV, and SR

**FIGURE 4:**
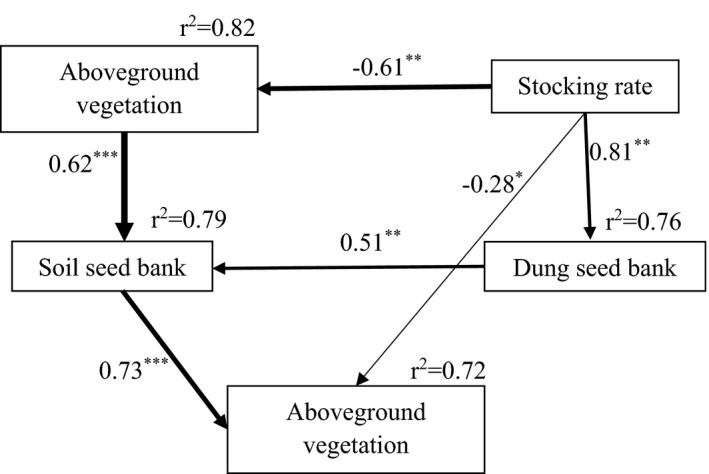
Structural equation models (SEM) showing the relationships between seed banks (SSB and DSB, 2016) and AGV (2016 and 2017). Species numbers (i.e., richness) were used for the calculations. The upper‐left box represents 2016 AGV, and the bottom‐center box represents 2017 AGV. Numbers on the arrows are standardized path coefficients that indicate the effective size of each relationship. Arrow width is proportional to the strength of the relationship. The proportion of explained variance is given as *r* ^2^. *p < .05, **p < .01, ***p < .001. Results of model fitting: *χ* ^2^ = 6.54; df = 4; *p* = .16. SSB: soil seed bank; DSB: dung seed bank; AGV, aboveground vegetation; SR, stocking rate

In 2016, SR had a significantly negative direct effect on AGV, and a significantly positive direct effect on the DSB (standardized path coefficients of −0.61 and 0.81, respectively; *p* < .05; Figure 4). The AGV had a significantly positive direct effect on the SSB (0.62; *p* < .001). In 2017, the SSB had a significantly positive direct effect on AGV (0.73; *p* < .001), and the DSB had a significantly positive direct effect on the SSB (0.51; *p* < .01). In addition, in 2017, SR had a significantly negative direct effect on AGV (−0.28; *p* < .05).

## 4 DISCUSSION

### 4.1 Effect of SR on AGV diversity

In 2016, there was no significant difference in the dissimilarity index for species composition among grazing paddocks. However, in 2017, the largest dissimilarity occurred among plots at SR 5.3 (Figure 2), which is consistent with the intermediate disturbance hypothesis that a hump‐shaped pattern exists between community diversity and disturbance (Catford et al., 2012; Connell, 1978; Grime, 2006). Bagchi and Ritchie (2010) found that the Bray–Curtis dissimilarity index for plant species composition was higher in grazed plots than in ungrazed plots in watersheds of the Trans‐Himalayas of northern India, which is similar to our result. Moreover, Navie and Rogers (1997) showed that high livestock pressure favors annual plant species that tolerate or avoid intense grazing and trampling through adaptations such as a prostrate growth habit and small seeds, which are more easily buried. In fact, grazing by large mammals could modify vegetation structure, composition, and function (Peco et al., 2005; Wang, Manuel, et al., 2019; Wang et al., 2018). Grazing impacts the AGV community through the removal of plant parts from groundcover vegetation and damage by trampling (Sanou et al., 2018). In addition, grazing creates a high‐light environment with multiple regeneration niches, and thereby assists in maintaining higher plant species richness (Hu et al., 2019; Milchunas & Noy‐Meir, 2002). Based on these and our own findings, it is likely that continued heavy grazing pressure has the potential to make annual species (e.g., *Eragrostis pilosa* (L.) Beauv., *C*. *glaucum*, and *Lappula deserticola* C. J. Wang) dominant on the Loess Plateau (The density of annual species increased in high SR treatments, Wang et al., Unpublished observations).

### 4.2 Similarities between seedlings and AGV richness

Our results indicated a weak relationship between the SSB and AGV richness across all treatments (Figure 3a). This may be due to relatively low seed production and seedling survival in harsh semiarid environments (Roberts, 1981), resulting in fewer active seeds in the SSB.

Due to differences in grazing regime, environmental factors, and spatial species distribution, the effect of grazing on the similarity between the SSB and AGV is under debate (Agra & Ne'eman, 2012; Peco et al., 1998; Ungar & Woodell, 1996). In this study, the low similarity between the SSB and AGV richness has been linked to the effects of grazing disturbance, including decreased floral abundance, which reduces SSB deposits. Thus, certain species could not be found in the SSB or failed to emerge. Such dissimilarity can also be attributed to AGV’s relatively rapid succession compared with the SSB (Karlík & Poschlod, 2014) and is consistent with the findings of Tessema et al. (2016), who reported a low mean similarity between the SSB and AGV in response to heavy grazing.

In 2016 and 2017, the similarity indices between the DSB and AGV richness were also low under different SR (Figure 3b). The relationship between the DSB and AGV is influenced by the selective feeding of livestock (Bagchi & Ritchie, 2010), physical and chemical properties of feces (Milotić & Hoffmann, 2016), and microhabitat properties at seed discharge sites (Calviño‐Cancela & Martín‐Herrero, 2009). Interestingly, in this study, seeds of c. 35% of pasture species germinated successfully after passing through the sheep gut. In semiarid environments such as the Loess Plateau, dung pellets provide significant protection for seed dispersal and survival until sufficient precipitation returns (Wang, Hu, et al., 2019); this is an adaptation for surviving in this type of harsh environment.

The maximum coefficient of similarity between the SSB and DSB (0.55 at 2.7 sheep ha^–1^; Figure 3c) indicates that the highest similarity occurs under light grazing pressure. The underlying mechanisms for this phenomenon were unclear. However, we found a positive correlation between the DSB and SSB (SEM: *r* ^2^ = 79%; Figure 4), which confirms that the DSB contributes to the SSB (Wang, Hu, et al., 2019).

### 4.3 Effect of grazing on SSB and DSB

Tan sheep grazing significantly increased the SSB species richness by changing the composition of the AGV (Table 2; Figure 4). However, there is contradictory evidence of grazing's impact on the SSB’s size, species richness, and composition (Kinloch & Friedel, 2005). Grazing impact is strongly shaped by historical grazing intensity and species‐specific sensitivity to grazing disturbance. Grazing reduces seed production through the consumption of flowers and immature seeds, or by reducing a plant's ability to produce seeds through biomass removal and physical damage from trampling (Hoshino et al., 2009; Paruelo et al., 2008; Sternberg et al., 2003). Consequently, intensive grazing can reduce the contribution of seeds to the SSB. As natural vegetative propagation is strongly associated with nearby biotic assemblages, it is reasonable to expect grazing to alter the SSB (Kinucan & Smeins, 1992). Moreover, grazing can promote the release and deep burial of seeds through trampling and disturbance by livestock, especially for small‐seeded species (Navie & Rogers, 1997).

Grazing livestock are significant endozoochorous seed dispersal vectors in grasslands (Gokbulak & Call, 2004; Oveisi et al., 2020). Livestock manure contains a high density of germinable seeds (e.g., 1.38 seeds·g^–1^ dry yak dung (Yu et al., 2013); 0.80 seeds·g^–1^ dry sheep dung (Kuiters & Huiskes, 2010); and 280–525 seedlings·L^–1^ fresh horse dung (Cosyns & Hoffmann, 2005)). In this study, the average seed density was 0.72 seeds·g^–1^ dry sheep dung, which is lower than previous locally determined values (Malo, 2000). In our study, eight, seven, and six plant species respectively germinated at SR 2.7, 5.3, and 8.7, indicating that the number of germinated species decreased with increasing SR (Figure 4). We attribute this to the low number of seeds remaining on reproductive plant culms as grazing pressure and disturbance increase (Navie & Rogers, 1997), which in turn reduces the probability of consuming mature seeds.

It should be noted that seeds egested in dung contribute significantly to the SSB (SEM: *r* ^2^ = 81%; Figure 4). As sheep dung is usually hard and dry, the contribution of seed egestion depends on the rate and degree of dung decomposition, which is primarily influenced by livestock trampling (Liu et al., 2011). In a study of cold‐season grazing on the Loess Plateau (Chen, 2015), sheep dung decomposition was fastest at the highest SR, and slowest at the lowest SR. Precipitation is another factor affecting dung decomposition (Whitford et al., 1982). In arid environments, seedling emergence is primarily related to soil water availability (Winkel & Roundy, 1991). In our study site, >70% of the total precipitation occurs between July and September, during which time many seeds are transferred from accumulated dung into the soil; the increased soil moisture then promotes seed germination and plant establishment.

## 5 CONCLUSIONS

Under cold‐season grazing on the Loess Plateau, seeds are cycled from plants, to animals, to soil. We found that SR was inversely correlated with seed cycling, which was depressed at higher grazing intensities. The cyclical nature of seed dispersal and propagation is a plant adaptation for survival in harsh, semiarid environments. Sheep grazing disturbance increases seed bank diversity, and assists in propagating and regenerating vegetation on the Loess Plateau and in other semiarid regions of the world.

## CONFLICT OF INTEREST

None.

## AUTHOR CONTRIBUTIONS

**Shu‐Lin Wang:** Writing‐original draft (equal). **An Hu:** Investigation (equal). **Fu‐Jiang Hou:** Funding acquisition (equal).

### OPEN RESEARCH BADGES

This article has been awarded Open Data, Open Materials, Preregistered Research Designs Badges. All materials and data are publicly accessible via the Open Science Framework at https://figshare.com/s/0000ad62a82c250fd939.

## Data Availability

Data are available through Figshare (https://figshare.com/s/0000ad62a82c250fd939).
